# Study of Chronic Hepatopathy in Patients With Sickle Cell Disease

**DOI:** 10.4021/gr2009.12.1327

**Published:** 2009-11-20

**Authors:** Maha M. Maher, Amany H. Mansour

**Affiliations:** a Internal Medicine Department, Gastroenterology Unit, Mansoura University 35516, Mansoura, Egypt; b Clinical Pathology Department, Mansoura University, Mansoura, Egypt

**Keywords:** Chronic hepatopathy, Sickle cell disease

## Abstract

**Background:**

Hepatic lesions in sickle cell disease were studied essentially in autopsy specimens. We investigated chronic hepatopathy in living adults with sickle cell disease and report the clinical, biochemical, and hepatic histological findings in these patients.

**Methods:**

A total of 170 adult patients with sickle cell syndrome were prospectively investigated. Clinical and laboratory investigation including liver function tests, serological tests for viral hepatitis, autoimmune hepatitis, and abdominal ultrasonography were performed in all of the patients. Liver biopsies were studied from 27 patients.

**Results:**

There was clinical evidence of jaundice in 123 (72.4%) patients, 118 (69.4%) patients had palpable liver, and 69% percent of the patients had elevated enzymes. Serological tests demonstrated the presence of hepatitis B infection in 18 (10.6%) patients and hepatitis C infection in 39 (23%) patients, serological markers for autoimmune hepatitis were positive in two female patients, one diagnosed chronic intrahepatic cholestasis. All 27 biopsies presented some degree of sickling hepatopathy. Moderate or marked liver siderosis was associated with the number of transfusions.

**Conclusions:**

The clinical spectrum of sickle cell disease ranges from mild liver function test abnormalities to significant hepatic abnormalities with marked hyperbilirubinemia. Multiple factors may contribute to the etiology of the liver disease, including ischemia, transfusion related viral hepatitis, iron overload, and gallstones.

## Introduction

Sickle cell disease (SCD) is a common genetic disorder which represents a major medical problem in certain parts of the world. It is characterized by chronic haemolytic anaemia and vaso-occlusive crises, which can lead to widespread vascular occlusion by sickled red blood cells leading to multiple organ infarctions. In this respect, SCD can be considered as a multisystem disease [1].

The liver can be affected by a number of complications due to the disease itself and its treatment [2, 3]. The risk of cholelithiasis, choledocholithiasis and liver failure increases in these patients due to sickling. In addition, viral hepatitis and other hepatobiliary diseases can also occure [4, 5]. There are more rare complications related to the sickle cell state: autoimmune hepatitis, hepatic infarction, hepatic abscess, hepatic biloma, Budd-Chiari syndrome, hyperammonaemia due to zinc deficiency [6].

Most pathologic studies of liver disease in sickle cell anemia and its variants were performed retrospectively on autopsy specimens, and, because of the prominent histologic features of intrasinusoidal sickling and Kupffer cell erythrophagocytosis, hepatic dysfunction was attributed to the intrahepatic sickling of erythrocytes in this hemoglobinopathy. Evidence of liver disease in sickle cell disease is obtained either from abnormal biochemical tests or postmortem liver biopsy specimen rarely an antemortem liver specimen [7]. Much of the literature on sickle cell hepatopathy lacks depth, because there are few sizeable or controlled studies [2].

The present study aimed to investigate chronic hepatopathy in living adults with sickle cell disease and to report the clinical, biochemical, and hepatic histological findings in these patients.

## Patients and Methods

The study was prospectively conducted from June 2007 to December 2008. Of 170 adult patients with sickle cell syndrome who admitted to King Fahd hospital (a major tertiary hospital in eastern region) KSA, only patients who were in the steady state were selected.

A general examination included assessing of the sclera for jaundice, enlargement of the liver and stigmata for chronic liver disease were done on all the patients before blood samples were taken for biochemical and coagulation studies. The number of transfusions received by each patient and alcohol or drug abuse was accurately determined. Demographic and Clinical characteristics of the study group are shown in Table1.

The liver enzymes, bilirubin, prothrombin time (PT) and Activated Partial Thromboplastin time (APTT) were done. Hepatitis B serology profile anti-HCV by a third-generation enzyme-linked immunosorbent assay, a third-generation RIBA for HCVand the polymerase chain reaction was applied to patients with HCV for virus quantification. Antinuclear antibody (ANA), Anti–smooth muscle antibody (ASMA) Anti–liver-kidney microsomal antibody (anti–LKM-1), and serum ferritin were done.

Abdominal ultrasound evaluation was performed in all patients, liver ultrasonographic changes reported were hepatomegaly, and cirrhosis. Abdominal ultrasound was also used to define patients with cholelithiasis.

A total of 27 patients satisfied the following inclusion criteria for hepatic biopsy and who agreed to give consent for liver biopsy; HBsAg positivity with altered results on hepatic function tests performed on two different occasions with an interval of at least 1 month between determinations; anti-HCV positivity confirmed by recombinant immunoblot assay (RIBA); and patients who underwent abdominal surgery, cholecystectomy, or splenectomy during the study period.

Hepatic specimens were fixed in 10% saline formalin and stained with hematoxylin and eosin, Perls' stain for iron, diastase-PAS, reticulin, and Gomori's trichrome. The histopathologic analysis was performed by a single pathologist with no previous information about the patient. Chronic hepatitis was also evaluated histologically by the modified histological activity index [8, 9]. Siderosis was graded as absent (-), mild (+), moderate (++), or marked (+++).

### Statistical Analyses

Data was analyzed using Student’s t and chi-square tests. The clinical, laboratory and pathological data of patients were compared using the nonparametric Mann-Whitney test. The same test was used to assess associations of the grade of liver siderosis and the number of previous transfusions with the value of ferritin. Statistical significance was set at P values less than 0.05.

## Results

The mean age of the patients was 26.3 years (range, 17 - 46), 34.1% were female. There was clinical evidence of jaundice in 123 patients (72.4%). One hundred eighteen patients (69.4%) had palpable liver. Eleven patients (6.5%) had never received blood transfusion. No association was observed between the total number of transfusions and jaundice (p = 0.92). Data showen in Table 1.

**Table 1:** Demographic, Clinical and Ultrasonographic Characteristics of The Study Group

Characteristics	Number
Male/female	112/58
Age (mean ± SD)	26.3 ± 5.6
History of blood transfusion	
None	11
< 10	78
> 10	81
Jaundice	123
Hepatomegaly	118
Ultrasonographic changes	
Hepatomegaly	121
Cirrhosis	3
Cholelithiasis	90
Cholecystectomy	13

The mean (range) of Alanine transaminase (ALT), Aspartate transaminase (AST) and alkaline phosphatase (ALP) were 41.0 (12 - 93) IU, 57.5 (26 - 128) IU, 268.5 (43 - 941) IU, respectively, Table 2.

**Table 2:** Laboratory Parameters of the Study Group

Laboratory Parameters	No. of Patients
Abnormal liver function tests	
ALT (> 40 IU)	27
AST (> 45 IU)	73
Bilirubin (> 2 mg/dL)	123
conjugated	60
ALP (> 200IU)	104
Albumin (< 3 gm/dL)	0
Prothrombin time (sec.)	19
Ferritin (ug/L)	
< 200	43
200 – 1000	69
> 1000	58
HbsAg (+)	18
HCV (+)	39
anti-LKM-1	0
ANA	2
ASMA	2

ALT: Alanine aminotransferase; AST: Aspartate aminotransferase; ALP: Alkaline phosphatase; Antinuclear antibody (ANA); Anti–smooth muscle antibody (ASMA); Anti–liver-kidney microsomal antibody (anti–LKM-1).

The gender or age of the patients did not significantly affect the level of the three enzymes. There was significant association between the liver size and elevation transaminases. Thirty-one percent of the patients had normal enzymes while 21% had the three enzymes deranged, 15.9%, 43% and 61.2% had abnormal ALT, AST and ALP respectively. The mean (range) of total bilirubin was 4.6 (0 - 31) mg/dL while for conjugated it was 3.1 (0 - 22.3) mg/dL. Only 18% and 5% had deranged PT and APTT respectively. The reference values for PT and APTT are 12 - 14 seconds and 30 - 40 seconds respectively.

Ferritin levels ranged from 61 to 8,134 ng/mL and were associated with the number of previous transfusions (above or below the median; Mann-Whitney, P = 0.0001). No association was observed between ferritin levels and the presence of viral hepatitis (P = 0.41).

Serological tests demonstrated the presence of hepatitis B infection in 18 (10.6%) patients and hepatitis C infection in 39 (23%). Hepatitis C serologies showed a relationship with the mean number of units transfused (p < 0.05) while hepatitis B did not (p = 0.88). Serological markers for autoimmune hepatitis; antinuclear and anti-smooth muscle antibodies were positive in two female patients. Abdominal ultrasound revealed hepatomegaly in 121 (71.2%) patients. cirrhosis in 3 (1.8%) and gall stones in 90 (53%) patients.

Biopsies were obtained from 27 patients (17 males and 10 females), ten of them with HCV and twelve with HBV, the remaining five patients underwent biopsy during splenectomy and cholecystectomy. The number of blood transfusions was determined in all patients. The median number of transfusions was 27.6 (ranged 11–91 blood units).

All 27 biopsies presented some degree of sickling hepatopathy (3 severe, 12 moderate and 12 mild), the main pathological features were Kupffer cell hyperplasia (27/27), 11 showed ischemic necrosis, 24 intrahepatic sickling and eight erythrophagocytosis were seen and did not correlate with liver disease.

Patients with HCV showed a histopathologic picture of chronic hepatitis; portal inflammation with formation of a lymphoid follicle, piecemeal necrosis and focal hepatocyte necrosis. A mild mononuclear inflammatory infiltrate was always present in correspondence to necrosis foci. All patients presented sinusoidal congestion, hypertrophy and hyperplasia of Kupffer cells, and pseudoacinar regeneration. Mild portal fibrosis was observed in two patients. The histologic activity index was 2 to 4 (inflammatory grading) and 0 to 2 (fibrotic staging).

Patients with HBV showed a histopathologic picture of incipient cirrhosis in three patients. The necro-inflammatory activity was shown by the presence of piecemeal necrosis, and focal hepatocyte necrosis. The portal tracts contained a mononuclear inflammatory infiltrate with the formation of lymphoid follicles. Mild sinusoidal congestion and dilatation were present, hyperplasia and hypertrophy of Kupffer cells, and pseudoacinar regeneration. The histological activity index was 7 and 10 (inflammatory grading) and 5 and 6 (fibrotic staging).

Moderate or marked liver siderosis was associated with the number of transfusions. Whereas nine patients with marked or moderate histological iron overload had received more than the median number of transfusions, patients with absent or mild iron overload received transfusion less than the median (P = 0.0001). If only patients without viral hepatitis are considered, the association remains significant (P = 0.003). Detailed histopathologic findings of patients with sickle cell disease are shown in Table 3.

**Table 3:** Histopathologic Findings of 27 Patients With Sickle Cell Disease

Patient NO	Kupffer cell hyper-plasia	Sinusoidal dilatation	Sickling RBC	Erythroph-agocytosis	Chole-stasis	Monouclear infiltration	Ischemic necrosis	Portal fibrosis	Hemoseder-osis
1	+	++	+	No	-	-	-	yes	-
2	++	++	+	No	-	-	-	No	++
3	++	+	++	Yes	+	+	+	No	+
4	+++	++	+	No	++	+	+	yes	+
5	++	+	+	No	+	++	-	No	+++
6	++	+	+	No	-	-	-	No	-
7	+	+	+	No	-	-	-	No	+
8	+++	++	++	No	+	+	+	No	+
9	+	++	+	Yes	+	+	-	No	++
10	++	+++	+	Yes	-	+	+	yes	+
11	++	++	+	No	-	-	-	No	+++
12	++	+	++	Yes	-	-	+	No	+
13	+	++	+	No	+	+	-	No	+
14	+	+	-	No	+	-	-	No	++
15	++	+++	++	No	+	+++	++	No	+
16	+	+	-	No	-	-	-	No	++
17	+	++	+	No	-	+	-	No	-
18	+++	+	+	No	+++	-	+	No	+++
19	+	++	+	No	-	+	—	No	++
20	++	+	+	No	-	+	-	No	+
21	++	+	+	Yes	+	+	-	yes	-
22	++	+++	++	Yes	++	-	++	No	+
23	+	+	+	No	-	-	-	No	+
24	++	++	+	Yes	+	+	+	No	+
25	+	++	+	No	-	-	+	No	+
26	+	++	+	Yes	+	-	+	No	+
27	+	+	-	No	-	-	-	No	++

One patient diagnosed as chronic intrahepatic cholestasis; he was deeply jaundiced, dark urine and severe pruritis, liver edge was 7 cm below costal margin, there was no stigmata of liver cell failure or chronic liver disease, bilirubin 31 mg/dL,72% conjugated, alkaline phosphatase 402 IU. Serological tests for hepatitis A, B and C were negative, no autoantibodies were detected. Abdominal ultrasound showed no dilatation of extrahepatic biliary dilatation. Liver biopsy showed maintained basic architectural pattern with mild to moderate piecemeal necrosis, mild spotty necrosis, sinusoidal dilatation, mild to moderate inflammation and widening of the portal areas and cholestasis.

Liver biopsies for two female patients with positive immune markers revealed signs of chronic active hepatitis with cirrhosis associated with dilated sinusoids lined by lymphocytes and hyperplastic Kupffer cells.

## Discussion

Various disorders of the hepatobiliary system can occur in patients with sickle cell anemia. The risk of cholelithiasis, choledocholithiasis and liver failure increases in these patients due to sickling. In addition, viral hepatitis and other hepatobiliary diseases can also occur [2, 4, 5]. Although there are some theories about frequency and pathophysiology of liver disease in patients with sickle cell disease, etiology and pathological features of chronic liver disease occurring in sickle cell anemia are under debate [10].

The findings reported in the present study suggest a multifactorial etiology for liver disease in SCD patients, including factors associated with chronic hemolytic anemia (cholelithiasis), multiple transfusions (viral hepatitis and iron overload) and the sickling process itself. However, data points to importance of vascular changes and the significant participation of the sickling process in most of patients, as reported by Charlotte et al [11]. Our results also, suggested that vascular damage is an important cause of chronic hepatic alteration among patients.

In our study, 39 (23%) patients had hepatitis C positive serologies, nearly the same results as that of De Vault et al who studied 121 patients and found 20.7% to be HCV Ab+ [12].

Fewer data have been published on hepatitis B serologies in sickle cell disease. Johnson et al [13] found the prevalence of HBsAg, sAb or cAb to be 19% in both SS and SC patients. In our patients, 18 (10.6%) had positive HBsAg, but 18 % had HBcAb+ and 21.2% had HBsAb+. Hepatitis C serologies showed a relationship with the mean number of units transfused while hepatitis B did not, this result is in agreement with other study [14].

The occurrence of autoimmune hepatitis type 1 in SCD patients has been previously reported in a 28-year-old woman and in two 11-year-old children (one boy, one girl) [15, 16]. In our study, serological markers for autoimmune hepatitis were positive in two female patients; clinically both had jaundice and hepatomogaly, liver biopsy revealed signs of chronic active hepatitis with cirrhosis associated with dilated sinusoids lined by lymphocytes and hyperplastic Kupffer cells. Perisinusoidal fibrosis was present, Whether a pathophysiological link exists between SCD and autoimmune hepatitis remains to be determined.

A distinct clinical presentation of sickle cell intrahepatic cholestasis, and a syndrome characterized by progressive cholestasis in the absence of cirrhosis had been reported in a small number of cases. These cases are characterized by right upper quadrant pain, extreme elevation of bilirubin, striking elevation of alkaline phosphatase and variable elevation of transaminases, histological features: intracanalicular cholestasis, sinusoidal dilatation, kupffer cell hyperplasia, and erythrophacocytosis [17]. This clinical and histological picture of chronic intrahepatic cholestasis was reported in one case in the studied group.

Abdominal ultrasound in patients with sickle cell anemia may reveal gallstones and increased echogenicity of the liver and pancreas caused by iron deposition [11]. In our study group, gall stones were reported in 90 patients.

Liver biopsy findings have already been described in other studies [17-19], however, all of these previous studies included the description of liver biopsies performed during autopsy, the present study reports the findings of 27 liver biopsies in living patients with chronic liver abnormality.

The histological findings reported reinforce our clinical observation that chronic liver abnormalities seem to be a multifactorial, since liver biopsies revealed siderosis, viral-related hepatitis, cholestasis and also vascular changes. Interestingly, siderosis at liver biopsy was noted in all patients with clinical hemosiderosis. Histopathological findings in our study were in accordance with previous reports [20-24] stating that intrasinusoidal sickling and Kupffer cell erythrophagocytosis, these were seen in almost all of our patients.

In conclusion, the clinical spectrum of SCD ranges from mild liver function test abnormalities in asymptomatic patients, to significant hepatic abnormalities with marked hyperbilirubinemia. Multiple factors may contribute to the etiology of the liver disease, including ischemia, transfusion related viral hepatitis, iron overload, and gallstones. SCD patients who present with increased liver enzyme activities, cholestatic jaundice and hypergammaglobulinemia, should be evaluated for autoimmune hepatitis as part of a full diagnostic workup. Continuous hepatic evaluation including periodical liver function tests, serological tests, ferritin levels and abdominal ultrasound should be performed. Initiation of specific therapy, if indicated, for viral hepatitis or iron overload, and ‘anti-sickling’ treatments, may prevent chronic hepatopathy in this population. Liver biopsy may be helpful for treatment decisions.
